# Emerging trends in cannabis administration for women with chronic pain

**DOI:** 10.1002/mhs2.88

**Published:** 2024-09-24

**Authors:** Erinn C. Cameron, Kristine M. Jacquin

**Affiliations:** 1 Department of Psychiatry, Boston Medical Center, Boston University Chobanian & Avedisian School of Medicine, Boston, Massachusetts, USA; 2 Department of Psychiatry, Massachusetts General Hospital, Boston, Massachusetts, USA; 3 School of Psychology, Fielding Graduate University, Santa Barbara, California, USA

**Keywords:** cannabis administration, cannabis use, chronic pain, public health policy, women’s health

## Abstract

Cannabis use among women who experience chronic pain is on the rise in the United States. However, little is known about women’s motives and preferences for cannabis administration. The purpose of this study was to characterize cannabis use among women with chronic pain. This study examined self-reported forms of cannabis administration and preferred source of cannabis, frequency and quantity of use, and self-reported side effects, and type, level, and intensity of chronic pain among adult women in the United States. This study also compared women who use cannabis for chronic pain and those who do not across the level of chronic pain, length of chronic pain, and the number of types of chronic pain experienced. Participants showed a significant preference (60%) for using recreational cannabis to treat chronic pain but reported that medical cannabis was more effective. For participants who preferred medical cannabis 24.3% reported daily use, as compared to only 7.8% of recreational cannabis users. Smoking was the most common form of administration (62.1%), followed by edibles (25.3%), vaporizing in any form (7.4%), tinctures and concentrates (3.2%), and topicals (2.1%). Participants reported using 1–6 different forms of cannabis administration. Those who preferred smoking were significantly likely to use all other forms of administration. However, those who preferred alternatives to smoking were significantly likely to use all forms of administration except for smoking. Medical cannabis users preferred to obtain cannabis from a dispensary, while recreational users preferred to obtain cannabis from unlicensed sources. Additionally, participants who used cannabis for chronic pain reported a 74% reduction in past 30-day opioid use. Future research is needed to investigate the health effects associated with single and combined forms of cannabis administration for women with chronic pain. Results can inform educational and intervention programs, treatment development, content regulation of products, policy formation, women’s health research, and public health guidelines.

With increasing legalization and growing acceptance of cannabis use for both medical and recreational purposes in the United States, there have been widespread changes in cannabis availability, varieties and forms, delivery methods and administration technology, perceived risk and benefits, and motivations for use (Compton et al., 2018; Spindle et al., 2019). Coincident with cannabis policy reform, perceptions of harm associated with cannabis use have decreased and prevalence of use has increased. Recent surveys suggest that cannabis use among individuals above the age of 12 ranges from 2% to 5% worldwide to 17.5% in the United States (Substance Abuse and Mental Health Services Administration, 2020; United Nations Office on Drugs and Crime UNODC, 2019). The United Nations Office on Drugs and Crime UNODC, (2019) reported a 60% increase in worldwide cannabis use over the past decade, with one in 10 adult cannabis users reporting daily use (Kroon et al., 2019).

There are more than 15.9 million registered medical cannabis patients in the United States with chronic pain, the most reported reason for registration (Marijuana Policy Project, 2023). However, varying levels of efficacy, tolerability, and safety have been indicated for cannabis with certain forms of chronic pain (Fitzcharles, Baerwald, et al., 2016). Long-term and heavy cannabis use [defined as (near) daily use] can sometimes lead to significant adverse health effects, including functional impairment and cannabis use disorder (CUD; Archie & Cucullo, 2019; Kitchigina, 2021; Kroon et al., 2019) Research suggests that approximately 19.5% of lifetime cannabis users meet CUD criteria, with 23% presenting with severe symptoms and chronic pain populations having a higher risk of developing CUD (Hasin et al., 2020). Additionally, higher rates of psychopathology and CUD have been associated with medical use as compared to recreational use (Turna et al., 2020), and the prevalence of CUD is rising among adults, including pregnant women (Pacula et al., 2016). According to the *Diagnostic and Statistical Manual of Mental Disorders*, 5th Edition (*DSM-5*; American Psychiatric Association, 2013), the anchoring aspect of a CUD diagnosis must include “a problematic pattern of cannabis use leading to clinically significant impairment or distress.”

To reduce the likelihood of problematic cannabis as noted in the *DSM-5*, more evidence-based research is needed to provide a foundation for federally standardized dosages and content for all cannabis products used to treat chronic pain at the consumer level. MacCallum and Russo (2018) provide practical guidelines to practitioners who also need clarification on standard of care dosages and titration to clinically effective levels. Further, there is an urgent need to develop public health-focused regulatory strategies that mitigate the negative health consequences of cannabis use while retaining the availability of high-quality and therapeutically effective legal cannabis products.

## 1 | GENDER AND CANNABIS USE

Gender differences in cannabis use are prevalent and associated with clinically important health outcomes. However, there is limited research regarding cannabis use and women and female cannabis users are underrepresented in health research (Dahl & Sandberg, 2015; Martin-Willet & Bidwell, 2021). While men use cannabis at higher rates overall than women, women’s usage is increasing at a faster rate (Substance Abuse and Mental Health Services Administration, 2018). Women have also reported increased cannabis use for chronic pain associated with health problems that are more common in women, such as fibromyalgia, menstrual pain, and endometrial pain (Ryan-Ibarra et al., 2015). In addition, cannabis is the most used illicit drug among women of childbearing age and during pregnancy in the United States (Carr, 2023).

Further, men are more likely to use cannabis medicinally and seek treatment for cannabis use disorder, while women are quicker to become addicted and experience a faster onset of CUD (Cuttler et al., 2016; Ehlers et al., 2010; Fairman, 2016; Kerridge et al., 2018). Also, women report more severe withdrawal symptoms and a greater negative impact of withdrawal and are more likely to have cannabis-related medical problems than men (Herrmann et al., 2015; Sherman et al., 2017). Women with substance use disorders also experience more severe adverse consequences in medical, psychiatric, and functional consequences than men (McHugh et al., 2018). Additionally, sexual minority women report higher rates of substance abuse problems than their heterosexual peers (Hughes, 2011; Lehavot & Simoni, 2011). While past work has explored demographic characteristics and cannabis use preferences, many of these studies used data before recent changes in legalization (Carliner et al., 2017; Hasin et al., 2020; Terry-McElrath et al., 2017). Therefore, it is important to investigate the sociocultural factors that may differentially influence women’s cannabis use patterns and behaviors. More recent data that includes gendered-usage patterns is necessary as the political and legal landscape regarding cannabis use continues to evolve rapidly.

## 2 | CANNABIS ADMINISTRATION

Route of administration (ROA) for cannabis often refers to the overall way that cannabis is delivered into the human body, such as through inhalation, oral-mucosal/sublingual, or topical and transdermal applications (Bruni et al., 2018). Delivery method often refers to the specific method used to deliver cannabis, such as smoking, edibles, vaporization, tinctures, topical applications, and oil extracts (Bruni et al., 2018). The term cannabis administration will be used as an umbrella term, encompassing both ROA and delivery method for this paper. Along with legalization changes, a multibillion-dollar cannabis industry produces high-potency herbal cannabis products, a growing number of boutique species, and novel delivery methods such as the e-pen vaping device. Higher concentrations of THC, averaging above 60%, available as high potency concentrates via the e-pen vaping device and various extracts, are more harmful in terms of addiction potential, psychosis, and cognitive impairment (Davenport, 2021; El Sohly et al., 2016; Englund et al., 2017; Murray et al., 2016). Although legalization has led to rapid growth, there is limited research regarding how these factors have affected perceptions of risks and benefits and patterns of cannabis use (Russell et al., 2018), especially for women.

Some adverse side effects of cannabis use may be prevented by using less harmful forms of administration while still preserving the identified therapeutic effect (Tashkin, 2017). For example, smoking cannabis has been associated with acute and chronic bronchitis and other respiratory problems (Loflin & Earleywine, 2015; Tashkin, 2017; Tetrault et al., 2007). Some medical professionals have discouraged use of combusted cannabis inhalation and instead recommend forms of administration that do not involve smoking, such as vaporization (Fischer et al., 2017; Loflin & Earleywine, 2015). Further, individuals who use traditional forms of vaporizations to consume cannabis have reported fewer adverse respiratory symptoms than those who predominantly smoke cannabis (Loflin & Earleywine, 2015).

Some research has indicated that individuals who use medical cannabis utilize vaporization or ingestion methods significantly more than those who use cannabis recreationally, with those who use recreationally more likely to utilize inhalation methods (Sznitman, 2017). However, other research has indicated that 92% of individuals who use cannabis recreationally and 82% of those who use cannabis medically reported smoking cannabis flower the last time they used cannabis (Pacula et al., 2016). Some studies have shown medical cannabis users prefer non-inhalation forms, such as tinctures, edibles, and topicals, which may avoid the hazards commonly associated with inhalation and have a slower time to onset, less euphoria, and longer-lasting effects (MacCallum & Russo, 2018). Self-reported reasons for choosing vaporization or oral forms of administration include fewer perceived health risks and stronger subjective drug effects as compared to smoking (Lee et al., 2016).

While just under half of adults and over half of adolescents report consuming cannabis edibles (Knapp et al., 2019; Schauer et al., 2016; Steigerwald et al., 2018), there are documented inconsistencies and inaccuracies in product labeling regarding THC and CBD content in edibles (Cao et al., 2016; MacCoun & Mello, 2015; Vandrey et al., 2015). Further, medical cannabis dosage for edibles has not yet been specified by the U.S. Food and Drug Administration due to its Schedule 1 status (FDA; Barrus et al., 2017). Additionally, higher rates of edible consumption have been associated with adverse psychiatric and cardiovascular outcomes (Health Canada, 2018) and increased risk for CUD (Cerda et al., 2019). Research also suggests that higher concentrations of THC, which are available as high potency concentrates for consumption via a vape pen or e-cigarette device, are more harmful in terms of addiction potential (including CUD), psychosis, and cognitive impairment (Cerda et al., 2019; El Sohly et al., 2016; Englund et al., 2017; Murray et al., 2016).

While research has indicated that men use a greater number of forms of cannabis administration than women (Baggio et al., 2014), women are increasingly being targeted with advertising for feminized versions of cannabis products, such as vaginal suppositories and topical products, and claims for symptom relief that are not supported by empirical evidence. This lack of empirical evidence supporting safety and efficacy for such products may lead to unintentional harmful cannabis use by women. Additionally, due to socio-cultural norms, women may choose more “feminine” cannabis products or forms of administration, even when those products lack established evidence for safety, tolerance, and efficacy.

## 3 | THE PRESENT STUDY

The overarching aim of the present study was to investigate emerging trends regarding form of cannabis administration, frequency and quantity of use, preferred source of cannabis, self-reported side effects, and perceived harm for medical and recreational cannabis for women with chronic pain. We also aimed to compare women who use cannabis for chronic pain and those who do not across the level of chronic pain, length of chronic pain, and the number of types of chronic pain experienced. Understanding women’s motives for selecting different forms of cannabis administration for symptom relief may have important implications for medical cannabis efficacy, cannabis policy development, chronic pain treatment, future patient and provider education, as well as innovations in women’s health research.

## 4 | MATERIALS AND METHODS

### 4.1 | Participants and recruitment

Participants were adults residing in the United States, fluent in English, who self-identified as women and reported having experienced chronic pain for at least half of the days for the previous consecutive 3 months. Participants were asked if they had consumed any form of cannabis in the past 30 days. Participants were recruited through advertisements placed on Facebook and Instagram groups aimed at women with chronic pain in April and May of 2021. Participants could elect to enter a drawing for one of five $50 gift cards. Qualtrics was used for survey delivery. Participants were required to answer all survey questions. Measures to ensure validity of responses included implementation of Qualtrics’ bot-detection feature and multiple validity/attention check questions. Institutional ethics approval was obtained from the Fielding Graduate University internal review board, and participants were provided informed consent.

### 4.2 | Survey

Participants were asked to complete several questionnaires that asked about sociodemographic characteristics, medical symptoms, chronic pain, cannabis use, preferences for cannabis administration, preferences for source of cannabis, perceptions about harm from cannabis, and frequency and quantity of cannabis use. The authors note that, in the United States, there are no current standardized descriptions for the terms “medical cannabis“ and “recreational cannabis.“ Participants were instructed that the term “medical cannabis“ referred to cannabis obtained by prescription, including derivative and synthetic versions available at the time of this study such as dronabinol, nabilone, Sativex, Epidiolex, and generic THC in oral or inhaled solutions often used in synthetic forms of cannabis products used to treat chronic pain (Le Boisselier et al., 2017). Participants were further instructed that “recommended” quantity, frequency, and type of cannabis referred to recommendation from a medical professional, from a prescription, or the recommended dosage and frequency stated on the product packaging… Participants were informed that they were not to report over the counter CBD products or cannabis derivatives derived from hemp products. The survey can be made available upon request by contacting the authors.

### 4.3 | Measures

#### 4.3.1 | Patient questionnaire

The patient questionnaire was comprised of three parts. Part 1 asked questions about demographic information such as age, gender, sexual orientation, marital status, income, education level, and occupational status. Part 1 also asked questions about type, severity, and duration of chronic pain symptoms. An example question is “How long have you experienced chronic pain?” (1 = *3–6 months* to 5 = *more than 10 years*). In Part 2, participants were asked about cannabis usage patterns such as, “Do you use any kind of cannabis to treat chronic pain symptoms?” (1 = *yes* or 2 = *no*).

In Part 3, participants were asked if they used medical or recreational cannabis, frequency of use, preferences regarding form of cannabis administration, choice of cannabis species, and how and where they obtain cannabis (source). While Part 3 asked questions about both medical and recreational cannabis use, participants only answered questions about the type of cannabis they self-reported using. An example question is “For medical cannabis use, how important are the following when choosing a delivery method?” using a scale of 1 to 4 (1 = *not at all important*, 2 = *somewhat important*, 3 = *important*, 4 = *very important*) with options such as duration of effect, time to onset of effect, ease of use, accessibility, symptom relief, number of side effects, level of perceived harm, cost, peer influence, % of THC, and % of CBD. This question was adapted from the Personal Importance of Mode of Delivery scale further described below (Shiplo et al., 2016b). Adaptation included adding additional delivery methods for cannabis use. The same question was asked about recreational cannabis use. Another example question is “Where do you get your medical cannabis?” (a dispensary, online, grow it myself, from another unlicensed source (e.g., friends, family, dealer), or don’t know). An additional question asks about the level of overall perceived harm from using medical or recreational cannabis (1 = *not all harmful* to 5 = *very harmful*).

#### 4.3.2 | Perceptions of personal importance of mode of delivery measure (PIMD)

The PIMD (Shiplo et al., 2016b) asked questions about an individual’s perceptions and personal importance regarding choice for form of cannabis administration. Questions were adapted to reflect cannabis delivery methods most commonly used in the United States. Participants were asked to rate 10 dimensions separately for each of the six listed forms of administration- smoking, vaporizer, vape pen/e-cigarette, edibles, tinctures and concentrates, and topical preparations. For each delivery method, the following question is asked “Compared to other ways of using cannabis, please rate *delivery method* on the following factors.” Example responses include duration of effect (1 = *very short* to 5 = *very long*), time to onset of effect (1 = *very slow* to 5 = *very fast*), the amount of cannabis needed to obtain the desired effect (1 = *very low* to 5 = *very high*), symptom relief (1 = *none at all* to 5 = *complete relief from symptoms*), and cost (1 = *very low* to 5 = *very high*). No reliability or validity data was provided for this measure (Shiplo et al., 2016a). Total score on each question was used to compare personal preference between forms of administration.

### 4.4 | Data analyses

SPSS version 26 and JASP version 0.17.3 were used to analyze data (IBM Corp., 2019; JASP Team, 2023). The assumptions of normality, homoscedasticity, linearity, and absence of multicollinearity were met. Descriptive statistics and frequencies were used to understand the participant sample. Hypothesis testing and identification of covariates was conducted using Pearson’s and bivariate correlations, multiple linear regression, ANOVA, Kruskal– Wallis H test, and chi-squared analyses.

## 5 | RESULTS

### 5.1 | Participant characteristics

The sample (*N* = 276) was primarily heterosexual (67.8%), White (74.6%), single (44.9%), attended at least some university or obtained an undergraduate degree (48.6%) or graduate degree (22.8%), employed full-time (50.00%), and age (*M* = 31.6), with 44.3% of single women reporting using cannabis, compared to 55.7% of women who were married or in a domestic partnership (see Table 1). Most of the sample (*n* = 199) reported using cannabis for chronic pain, while 77 women reported that they did not use any form of cannabis. Women with more education were less likely to report using cannabis for any reason. Most women who reported using cannabis for chronic pain preferred recreational cannabis (*n* = 144) to medical cannabis (*n* = 55), while 67 women reported often using both types of cannabis. However, participants reported that medical cannabis was more effective in treating chronic pain. Additionally, participants who reported using medical cannabis to treat chronic pain reported that medical cannabis was legal in their state (54.3%), not legal (20.7%), or partially legal (16.3%). While participants who reported using recreational cannabis to treat chronic pain reported that recreational cannabis was legal in their state (31.2%), not legal (48.9%), or partially legal (9.4%). Additionally, participants who used any form of cannabis for chronic pain reported a 74% reduction in past 30-day opioid use.

The mean level of current pain intensity for the total study sample ranged 3-10 (*M* = 7.05, SD = 1.68) on a scale of 1–10 (1 = *least pain* to 10 = *extreme pain*). Bisexual women (*M* = 7.20, SD = 1.58) reported higher levels of chronic pain than heterosexual women (*M* = 7.17, SD = 2.00), and Hispanic/Latinx women reported the highest levels of chronic pain (*M* = 7.88, SD = 1.93). Back pain and lower back pain were the most reported types of chronic pain. Women who reported using cannabis for chronic pain (*M* = 6.95, SD = 1.71) reported lower levels of pain than those who did not use cannabis (*M* = 7.27, SD = 1.58). See Table 1 for participant characteristics and Table 2 for descriptive statistics for chronic pain variables.

### 5.2 | Chronic pain

See Table 2 for chronic pain length, level, type, and the number of types of chronic pain reported by participants. The following analyses compare women who use cannabis for chronic pain and those who do not across the level of chronic pain, length of chronic pain, and the number of types of chronic pain experienced. A Kruskal-Wallis H test showed a statistically significant difference in the number of types of pain between the those who use cannabis to treat chronic pain and those who do not. Those who use cannabis reported a higher number of types of chronic pain (*M* = 3.23, SD = 1.73) than those who do not use cannabis (*M* = 2.53, SD = 2.19), *F*(1,207) = 5.61, *p* = 0.019, Cohen’s *d* = 0.35, χ^2^(1) = 12.25, *p* < 0.001, with a mean rank score for the number of types of pain of 116.14 for those who used cannabis to treat chronic pain, and 85.90 for those who did not use cannabis to treat chronic pain.

A Kruskal–Wallis H test did not indicate a statistically significant difference in the level of chronic pain between for those who use cannabis to treat chronic pain (*M* = 6.95, SD = 1.71) and those who do not (*M* = 7.27, SD = 1.67), *F*(1, 207) = 1.78, *p* = 0.184, Cohens *d* = 0.19, χ^2^(1) = 2.47, *p* = 0.116, with a mean rank score for level of chronic pain of 100.08 for those who used cannabis to treat chronic pain, and 113.44 for those who did not use cannabis to treat chronic pain.

## 6 | CANNABIS

### 6.1 | Quantity, frequency, and type of cannabis use

For medical cannabis, the majority of participants reported using the recommended quantity, with 24.3% reporting daily use (see Table 3). Point biserial correlations indicated a significant positive relationship between quantity of medical cannabis used (past 30 days) and employment status *r* = 0.395, *p* = 0.015. Pearson’s correlations further indicated a significant positive relationship between quantity of medical cannabis use and length of chronic pain, *r* = 0.369, *p* = 0.025.

For recreational cannabis, 58.8% reported using more than the recommended quantity with only 7.8% reporting daily use (see Table 3). Similar to medical cannabis, correlational analysis indicated a significant positive relationship between quantity of recreational cannabis used (past 30 days) and employment status *r* = 0.419, *p* = 0.009 and length of chronic pain, *r* = 0.276, *p* = .028.

## 7 | CANNABIS ADMINISTRATION

Overall, for participants who reported using any type of cannabis (*n* = 199), smoking was the preferred form of administration (62.1%), followed by edibles (25.3%), vaporizing in any form (7.4%), tinctures and concentrates (3.2%), and topicals (2.1%). Women who reported using any form of cannabis to treat chronic pain reported using from 1 to 6 different forms of administration (see Table 4).

### 7.1 | Comparisons across forms of cannabis administration

#### 7.1.1 | Smoking

Of those participants who reported **utilizing smoking** as a primary form of administration for cannabis, 77.8% said they also utilized vaping/vape-pen, and the relationship was significant χ^2^(1) = 6.041, *p* = 0.014. The following relationships were not significant, edibles (64.8%) χ^2^(1) = 1.060, *p* = 0.303, tinctures and concentrates (50%) χ^2^(1) = 1.288, *p* = 0.256, topicals (53.6%) χ^2^(1) = 0.776, *p* = 0.379, and vaporizers (70.0%), χ^2^(1) = 0.845, *p* = 0.358.

#### 7.1.2 | Vaping/e-pen

Of those participants who reported **vaping/e-pen** as their primary form of administration for cannabis all relationships with other delivery methods were significant, with 75.0% also utilizing a vaporizer χ^2^(1) = 26.42, *p* < 0.001, tinctures and concentrates (54.5%), χ^2^(1) = 9.54, *p* = 0.002, topicals (46.4%), χ^2^(1) = 6.26, *p* = 0.012, edibles (39.4%), χ2(1) = 10.78, *p* = 0.001, and smoking (35.4%), χ^2^(1) = 10.78, *p* = 0.001.

#### 7.1.3 | Edibles

Of those participants who reported **utilizing edibles** as their primary form of administration for cannabis, the following relationships with other delivery methods were significant, with 77.8% also utilizing vaping/vape-pen, χ^2^(1) = 10.776, *p* = 0.001, vaporizer (75.0%) χ^2^(1) = 3.962, *p* = 0.047, tinctures and concentrates (86.4%), χ^2^(1) = 10.769, *p* = 0.001, and topicals (71.4%), χ^2^(1)= 4.070, *p* = 0.004. The relationship with smoking (58.2%) was not significant, χ^2^(1) = 1.060, *p* = 0.303.

#### 7.1.4 | Topicals

Of those participants who reported **utilizing topicals** as their primary form of administration for cannabis, the following relationships with other delivery methods were significant, with 36.1% also utilizing vaping/vape-pen (36.1%), χ^2^(1) = 6.26, *p* = 0.012, vaporizer (40.0%) χ^2^(1) = 4.78, *p* = 0.029, tinctures and concentrates (54.5%), χ^2^(1) = 17.07, *p* < 0.001, and edibles (28.2%), χ^2^(1) = 4.07, *p* = 0.044. However, the relationship with smoking (19%) was not significant, χ^2^(1) = 0.776, *p* = 0.379.

#### 7.1.5 | Tinctures and concentrates

Of those participants who reported **utilizing tincture and concentrates** as the primary form of administration for cannabis the following relationships with other delivery methods were significant, with 36.1% also utilizing vaping/vape-pen (36.1%), χ^2^(1) = 6.26, *p* = 0.012, vaporizer (40.0%) χ^2^(1) = 4.78, *p* = 0.029, tinctures (54.5%), χ^2^(1) = 17.07, *p* < 0.001, and edibles (28.2%), χ^2^(1) = 4.07, *p* = 0.044. The relationship with smoking (19%) was not significant, χ^2^(1) = 0.776, *p* = 0.379.

## 8 | MOTIVATION

Participants were asked to rate reasons for choosing a form of cannabis administration on a Likert-type scale (1 = *least important to* 5 = *most important*). For both medical cannabis and recreational cannabis, participants rated peer influence as the primary reason, medical (*M* = 3.89, SD = 1.28), recreational (*M* = 4.05, SD = 1.15). The second highest mean ratings were % CBD (*M* = 2.93, SD = 1.41) for recreational cannabis and % THC (*M* = 2.60, SD = 2.20) for medical cannabis. For medical cannabis, level of perceived harm (*M* = 2.60, SD = 1.24) received the same mean rating as %THC. See Table 5.

### 8.1 | Level of belief that medical cannabis is harmful

Bivariate correlations indicated a significant positive relationship between the level of belief that medical cannabis is harmful and the number of cannabis delivery methods Spearman’s *r* = 0.440, *p* = 0.008, and significant inverse relationships with age, Pearson’s *r* = −0.387, *p* = 0.002, and length of chronic pain, Pearson’s *r* = −0.398, *p* = 0.018. No significant associations were found between the level of belief that medical cannabis is harmful and demographic characteristics, preferred form of cannabis administration, chronic pain intensity, or the number of types of chronic pain experienced.

### 8.2 | Level of belief that recreational cannabis is harmful

Bivariate correlations indicated significant positive relationship between level of education Pearson’s *r* = 0.203, *p* = 0.040, and significant inverse relationships with number of types of chronic pain experienced, Spearman’s *r* = −0.285, *p* = 0.004, number of forms of cannabis administration used, Spearman’s *r* = −0.202, *p* = 0.041, and length of chronic pain experienced, Pearson’s *r* = −0.217, *p* = 0.028. No other relationships were significant.

### 8.3 | Preferred source for obtaining cannabis

For women who reported using recreational cannabis for chronic pain, 62% (*n* = 90) preferred to obtain cannabis from unlicensed sources such as a friend, neighbor, family member, or dealer; while 19% (*n* = 27) preferred a licensed dispensary, and 19% (*n* = 27) preferred “other” source or to grow cannabis themselves. For women who reported using medical cannabis for chronic pain, 64% (*n* = 35) preferred to obtain cannabis from a licensed dispensary, while 22% (*n* = 12) preferred an unlicensed source, and 7% (*n* = 4) preferred online sources, and 7% (*n* = 4) preferred “other” source or to grow cannabis themselves.

## 9 | SELF-REPORTED SIDE EFFECTS

Participants reported feeling high as the primary side effect for both medical (32.73%) and recreational cannabis (71.20%). Other notable self-reported side effects for recreational cannabis were feeling quiet or disconnected (62.08%), increase in appetite (55.20%), dry mouth (48.8%), and drowsiness (48.00%). Other notable self-reported side effects for medical cannabis were dry mouth (29.09%), feeling quiet or disconnected (29.01%), and excessive thirst (20.00%). See Table 6 for all self-reported side effects.

## 10 | DISCUSSION

Using any form of cannabis to self-medicate for chronic pain is increasing among women in the United States. While there are many cannabis products currently being marketed for chronic pain, there are conflicting interpretations of the evidence regarding the efficacy, tolerability, and safety of cannabinoids for pain management, especially according to gender (Cameron & Hemingway, 2020; Fitzcharles, Ste-Marie et al., 2016). Understanding preferences and motives for choosing specific forms of cannabis administration is needed to help inform and target future educational and intervention programs, treatment development, content regulation of products, public health policy, and women’s health research. Further, investigating diverse methods of treating chronic pain in women is an urgent public health concern, particularly regarding the safety, efficacy, and tolerability of cannabis products.

### 10.1 | Women and chronic pain

Studies indicate that women are at a higher risk of chronic pain and have a lower pain threshold than men (Malon et al., 2018) and that chronic pain prevalence is 20% to 30% higher in women (Belfer, 2017; Haroutounian et al., 2016). In the present study, sexual minority women and those of Hispanic/Latinx heritage reported higher levels of chronic pain intensity, supporting past research indicating that ethnicity, cultural heritage, and marginalized identities may play a role in how chronic pain is experienced, conceptualized, and treated (Campbell & Edwards, 2012; Meints et al., 2019; Wallace et al., 2021). Further, for women who experience chronic pain, 50% of participants were fully employed with an overall reported mean income lower than the national average for the United States. These results align with research indicating that individuals with adverse physical health conditions, such as chronic pain, are more likely to have lower incomes and be under-employed (Groll-Prokopczyk, 2016). These individuals may lack access to medical insurance or healthcare and, therefore, may turn to alternative treatments for chronic pain, such as cannabis.

## 11 | CANNABIS USE FOR WOMEN WITH CHRONIC PAIN

While participants who reported using medical and recreational cannabis to treat chronic pain shared some characteristics, those who used medical cannabis were more likely to report daily use. Results are supported by research suggesting that medical cannabis users are more likely to endorse daily consumption than recreational cannabis users (Lin et al., 2016). Additionally, participants reported a 74% reduction in past 30-day opiate use. Research indicates cannabis use is widely associated with decreased prescription medication use, particularly opiates (Boehnke et al., 2019; Corroon et al., 2017; Denduluri et al., 2018; Reiman et al., 2017). Using cannabis as a replacement for prescription medications likely indicates that many individuals who use cannabis administer daily therapeutic doses over an extended period, making the therapeutic use of cannabis a significant public health concern. Additionally, less is known about those who self-medicate with cannabis for chronic pain since past public policy has posed significant barriers to studying unauthorized and nonmedical cannabis use. Therefore, additional public health guidelines are needed to provide more clear benefits and risks of long-term cannabis use for individuals with chronic pain.

In addition, for the current study, recreational cannabis users reported a higher prevalence of side effects, particularly regarding feeling high, feeling quiet or disconnected, increase in appetite, dry mouth, and confusion or forgetfulness. Medical cannabis users reported far less pronounced side effects, with no reports of vomiting or hallucinations. Further, while some past research has reported no sociodemographic differences between medical and recreational cannabis users (Woodruff & Shillington, 2016), present results indicate that recreational users are more likely to be younger and have lower incomes than those who use medical cannabis.

Further, while studies have suggested that women are more likely to purchase cannabis from nonpublic sources (Dahl & Sandberg, 2015; Hathaway et al., 2018), present results indicate that women preferred to purchase medical cannabis from a licensed dispensary and recreational cannabis from unlicensed sources. Additionally, several studies have cited cost as the main driver for most recreational cannabis purchases and percentage of CBD the top consideration for medical cannabis purchases (Shi et al., 2019), with recent work highlighting the lack of available data regarding other aspects that influence choice of cannabis source (Donnan et al., 2022). Present results indicated that women who use recreational cannabis for chronic pain did not consider cost an important factor, but those who use medical cannabis did consider cost to be a key factor. Further, overall, participants relied most heavily on peer influence when deciding where to purchase any kind of cannabis.

In addition, studies have indicated that cannabis use for women of reproductive age (15–49) may be influenced by socioeconomic status, age, and ethnicity (Beatty, 2012; Ko et al., 2015). Current results indicated significant relationships between several sociodemographic factors and cannabis use for women in this age group. Specifically, women who used cannabis for chronic pain reported lower levels of education than their peers who did not use cannabis. In addition, employment status was significantly correlated with the quantity of cannabis used, and women with higher incomes reported greater cannabis use overall.

## 12 | CANNABIS ADMINISTRATION

Present findings revealed that racial and sexual identity were covariates for choice of cannabis administration for women, indicating that these factors may play a role in women’s decisions regarding the choice of cannabis delivery method. Additionally, while some studies have suggested that individuals who use medical cannabis are more likely to utilize vaporization or ingestion than those who use recreational cannabis (Sznitman, 2017), other research indicates that smoking has traditionally been the most utilized delivery method overall (Pacula et al., 2016). Present results indicated that both medical and recreational cannabis users preferred smoking as their primary choice of administration. These results contradict some past research indicating that those who use medical cannabis are more likely to avoid delivery methods involving inhalation (MacCallum & Russo, 2018).

Present results also indicated that edibles are the second most prevalent choice for cannabis administration for women who use any form of cannabis. Studies have suggested that individuals may choose edibles as one way to avoid cannabis use stigma since edibles can be consumed more discreetly than many forms of inhalation (Barrus et al., 2017). However, edibles often have inconsistent and sometimes inaccurate content labeling for THC and CBD concentration and are metabolized differently than when cannabis is consumed via inhalation, primarily due to the delayed onset of drug effect with ingestion (Cao et al., 2016; MacCoun & Mello, 2015; Vandrey et al., 2015). Further, the FDA has yet to regulate medical cannabis dosage for edibles due to its Schedule 1 status (Barrus et al., 2017). There is an urgent need to more comprehensively understand how women with chronic pain utilize different forms of cannabis administration and the extent to which specific delivery methods are associated with differential cannabis use outcomes.

## 13 | LIMITATIONS

Although self-report measures are frequently utilized in many areas of social science research, the validity and accuracy of self-reporting are common limitations, particularly regarding construct validity and social desirability bias (Deshields et al., 1995). With societal bias against substance users, social desirability bias may have been a limitation of the present study. Additionally, research indicates that discrepancies between self-report cannabis consumption and accurate consumption rates are prevalent (El Marroun et al., 2011; Yonkers et al., 2011), making the determination of actual prevalence rates for cannabis use challenging. Future studies could utilize toxicology screening, such as urinalysis or hair follicle analysis, to determine rates of cannabis consumption more accurately. However, this approach is not practical with online survey research. Additionally, dosage and instruction labelling are not well standardized and may not be reliable; it is possible that participants were under or overestimating their actual use, especially if self-titrating to effective levels (MacCallum & Russo, 2018).

Additionally, study participants may have been using cannabis or other analgesics to control chronic pain during the present study, which may have affected reporting of current levels of pain intensity. However, asking study participants not to engage in treatment for chronic pain during the survey would have been unethical. Further, the subjective nature of pain makes it difficult to measure and compare pain ratings across participants (Kroenke, 2018).

## 14 | CONCLUSION

The full impact of increased legalization, such as rapidly evolving technology for cannabis administration and increased accessibility for medical and recreational cannabis for women, has yet to be realized. As cannabis use in all forms increases for women, future studies are urgently needed to identify not only how women experience chronic pain but also how and why women use cannabis to treat chronic pain. Available studies regarding gender and cannabis use indicate that, like other substance use, societal gender norms can influence cannabis use patterns. However, the current lack of knowledge regarding how women make decisions regarding cannabis use, primarily what drives the choice of preferred type, source, form of administration, quantity, and frequency for cannabis, is concerning. Future cannabis research should integrate sex and gender so that information regarding cannabis use can be tailored appropriately before being disseminated to health care providers and consumers. Such research is also needed to develop educational information for women so that they can make evidence-based choices about using cannabis to treat chronic pain.

Further, novel harm reduction, intervention, and prevention approaches are needed to address cannabis use among women who choose to self-medicate for chronic pain. Since current results indicate that women with intersecting marginalized identities may experience greater intensity and length of chronic pain, and similar factors influence how women experience chronic pain, addressing the social determinants of chronic pain is essential for realizing healthcare equity. Future studies are needed to understand better how gender intersects with other social determinants of health and cannabis use for women, such as sexual identity, ethnicity, education, and other socioeconomic factors.

Results of the current study can inform evidence-based policies for reducing problematic and unintentionally harmful cannabis use. Results can also support the development of regulations regarding required dissemination of cannabis use information, accurate package labeling, and mandatory continuing education for healthcare providers who prescribe or recommend cannabis. Such policies and regulations could facilitate measures to reduce population-level harm for women who use cannabis to treat chronic pain, such as regulating the potency and dosage recommendations of all forms of administration for medical and recreational cannabis.

## Figures and Tables

**TABLE 1:** Participant characteristics.

Study sample		Length of chronic pain *M*(SD)	Level of chronic pain *M*(SD)
Gender			
Female	96%	3.47 (1.70)	7.11 (1.66)
Nonbinary	3.3%	3.89 (1.62)	5.67 (1.88)
Other	0.4%	5.00 (1.41)	5.00 (1.41)
Sexual identity			
Heterosexual	67.8%	3.28 (1.69)	7.17 (1.58)
Bisexual	17.8%	3.73 (1.66)	7.20 (2.00)
Lesbian	2.5%	3.86 (1.22)	7.00 (1.29)
Pansexual	5.4%	4.40 (2.00)	5.71 (1.68)
Asexual	4.0%	3.67 (1.34)	5.83 (1.47)
Polysexual	0.4%	5.00 (0.00)	5.00 (0.00)
Preferred not to say	2.2%	5.83 (1.47)	3.67 (1.37)
Ethnicity			
White	74.6%	3.61 (1.71)	6.98 (1.56)
African American or Black	9.8%	2.81 (1.36)	7.11 (1.56)
Hispanic or Latinx	6.2%	3.06 (1.71)	7.88 (1.93)
Indigenous American or first nations	1.4%	2.00 (0.816)	6.75 (1.89)
Alaska native	1.1%	1.67 (0.58)	7.00 (0.00)
Pacific islander	0.7%	3.00 (0.00)	11.0 (0.00)
Mixed ethnicity	2.9%	5.00 (1.31)	5.75 (2.38)
Other	1.8%	4.60 (1.52)	7.40 (2.19)
Marital status			
Single	44.9%	3.09 (1.69)	7.19 (1.72)
Married	34.8%	3.99 (1.60)	6.89 (1.70)
Domestic partnership	15.6%	3.55 (1.66)	6.91 (1.60)
Divorced	2.2%	4.17 (1.72)	7.33 (1.21)
Separated	0.4%	9.00 (0.00)	4.00 (0.00)
Widowed	0.4%	7.00 (0.00)	2.00 (0.00)
Other relationship status	1.8%	7.05 (1.68)	3.50 (1.69)
Education			
Some university	25.4%	2.87 (1.55)	7.44 (1.37)
Undergraduate degree	23.2%	3.42 (1.70)	6.63 (1.79)
Graduate degree	22.8%	3.95 (1.68)	6.94 (1.56)
Highschool diploma	12.3%	3.41 (1.62)	7.32 (2.00)
Doctoral degree	7.2%	4.80 (1.01)	6.70 (1.69)
Trade or vocational degree	3.6%	4.3 (1.64)	8.00 (1.70)
GED	2.5%	3.14 (1.77)	7.32 (2.00)
Did not complete high school	2.2%	1.50 (0.840)	6.33 (1.97)
Other	0.7%	7.05 (1.68)	3.50 (1.69)
Employment status			
Full-time work	50%	3.14 (1.62)	7.23 (1.61)
Part-time work	14.5%	3.98 (1.72)	6.53 (1.47)
Unemployed or laid off	6.5%	3.72 (1.71)	7.17 (1.82)
Searching for work	4.0%	2.82 (1.54)	7.09 (1.82)
Homemaker	6.2%	3.65 (1.80)	6.94 (2.18)
Retired	3.3%	5.44 (0.88)	6.33 (1.58)
Out of work due to COVID-19	1.4%	3.75 (1.26)	6.00 (1.58)
Other	13.7%	3.86 (1.73)	7.17 (1.91)
Income			
<$5,000	15.6%	3.30 (1.82)	7.12 (1.78)
$5,000–$11,999	12.3%	3.50 (1.57)	7.29 (1.99)
$12,000–$15,999	8.0%	3.45 (1.57)	7.73 (1.42)
$16,000–$24,999	12.7%	3.31 (1.55)	7.17 (1.40)
$25,000–$34,999	11.2%	3.29 (1.58)	6.93 (1.62)
$35,000–$49,999	11.6%	3.19 (1.80)	6.78 (1.85)
$50,000–$74,999	10.1%	3.68 (1.63)	7.32 (1.59)
$75,000–$99,999	4.3%	5.17 (1.03)	6.25 (1.42)
>$100,000	2.5%	3.53 (1.67)	6.78 (1.60)
Don’t know/refuse to answer	11.6%	6.78 (1.60)	3.53 (1.67)

*Note*: *N* = 276.

**TABLE 2:** Chronic pain length, level, type, and number of types of chronic pain for the study sample.

	All participants *N* = 276 *M(SD)*	Cannabis use *n* = 199 *M(SD)*	Non-cannabis use *n* = 77 *M(SD)*
Length of chronic pain	3.50 (1.69)	3.73 (1.68)	3.33 (1.70)
3–6 months	14.5%	13.6%	15.6%
6–12 months	19.2%	12.1%	23.4%
1–2 years	18.1%	14.4%	19.5%
3–5 years	16.7%	23.5%	11.7%
6–10 years	13.4%	15.2%	14.3%
10+ years	18.1%	21.2%	15.6%
Number of types of pain	2.84 (2.02)	3.23 (1.73)	2.53 (2.19)
1	34.0%	18.9%	50.6%
2	17.0%	18.9%	15.6%
3	20.7%	27.3%	6.5%
4	13.4%	17.4%	11.7%
5	5.4%	6.8%	3.9%
6	2.9%	2.3%	3.9%
7	3.6%	4.5%	5.2%
8	1.1%	1.5%	0.0%
9	1.4%	1.5%	1.3%
10-13	0.4%	0.8%	1.3%
Types of chronic pain reported			
Abdominal pain	26.1%	16.7%	32.5%
Back pain	42.0%	53.0%	31.2%
Lower back pain	40.2%	47.0%	39.0%
Neck pain	29.3%	31.8%	27.3%
Cancer pain	1.1%	0.8%	1.3%
Chronic pain following surgery	5.8%	7.6%	3.9%
Menstrual pain	30.4%	36.4%	23.4%
Endometrial pain	7.6%	6.1%	7.8%
Vulvar pain	3.6%	4.5%	3.9%
Neuropathic pain	15.9%	18.9%	11.7%
Pelvic pain	7.2%	9.1%	6.5%
Chronic pain due to trauma or injury	10.1%	12.1%	9.1%
Fibromyalgia pain	12.3%	11.4%	15.6%
Multiple sclerosis pain	1.8%	3.0%	2.3%
Musculoskeletal pain	13.8%	18.2%	11.7%
Migraine pain	23.6%	26.5%	23.5%
Other types of chronic pain	12.7%	21.2%	5.2%
Pain intensity/level (1–10)	7.05 (1.68)	6.95 (1.71)	7.27 (1.58)
1- no pain	0%	0%	0%
2	1.1%	0.8%	0%
3	7.3%	7.6%	7.8%
4	12.4%	14.4%	10.4%
5	12.0%	15.9%	5.2%
6	24.9%	20.5%	23.4%
7	26.9%	24.2%	35.1%
8	10.9%	10.6%	14.3%
9	2.9%	3.3%	2.6%
10- extreme pain	2.5%	2.3%	1.3%

*Note*: Study Sample *N* = 276.

**TABLE 3:** Rceported quantity of medical cannabis and frequency of medical and recreational cannabis.

Medical cannabis: Quantity	
Less than recommended	38.9%
As recommended	50.0%
More than recommended	5.6%
A lot more than recommended	5.6%
Medical cannabis: Frequency	
More than once a month	37.8%
At least once a month	54.1%
At least once a week	13.5%
Almost every day	5.4%
Everyday	24.3%
I don’t know	2.7%
Recreational cannabis: Quantity	
Less than recommended	9.2%
As recommended	24.8%
More than recommended	58.8%
A lot more than recommended	7.2%
Recreational cannabis: Frequency	
More than once a month	43.1%
At least once a month	19.6%
At least once a week	3.9%
Several times a week	9.8%
Everyday	7.8%
More than once a day	5.9%
I don’t know	9.8%

*Note*: Medical Cannabis *n* = 55, Recreational Cannabis *n* = 144.

**TABLE 4:** Forms of cannabis administration reported.

Number Reported	
1	29.9%
2	30.8%
3	12.3%
4	8.5%
5	1.5%
6	4.6%
Frequency	
Smoking	61.4%
Edibles	54.6%
Vaping/e-pen	27.7%
Topicals	29.3%
Tinctures & concentrates	16.9%

*Note: n* = 199.

**TABLE 5:** Means and standard deviations of likert ratings for reported motive for choosing cannabis form of administration.

Motive	Recreational cannabis *n* = 144 *M*(SD)	Medical cannabis *n* = 55 *M*(SD)
Duration of effect	2.51 (1.26)	2.20 (0.994)
Time to onset of effect	2.66 (1.24)	2.20 (1.05)
Ease of use	2.14 (1.18)	2.20 (1.11)
Accessibility	2.14 (1.18)	2.00 (1.06)
Ability to obtain needed dose	2.40 (1.27)	2.03 (1.01)
Symptom relief	2.34 (1.37)	1.97 (1.07)
Number of side effects	2.48 (1.26)	2.46 (1.20)
Level of perceived harm	2.66 (1.27)	2.60 (1.24)
Cost	2.36 (1.16)	2.46 (1.20)
Peer influence	4.05 (1.15)	3.89 (1.28)
% THC	2.80 (1.40)	2.60 (2.20)
% CBD	2.92 (1.41)	1.19 (1.05)

**TABLE 6:** Self-reported side effects for medical and recreational cannabis.

Side effects	Medical cannabis	Recreational cannabis
Feeling “high”	32.73%	71.20%
Dry mouth	29.09%	48.8%
Feeling quiet or disconnected	29.01%	62.08%
Excessive thirst	20.00%	34.4%
Fatigue	18.18%	24.00%
Increase in appetite	16.36%	55.20%
Confusion/forgetful	16.36%	32.8%
Anxiety	16.36%	30.4%
Sweating	9.09%	9.6%
Loss of appetite	9.0.%	8.8%
Nausea	7.27%	16.8%
Paranoia	5.45%	25.6%
Dehydration	5.45%	10.4%
Blurred vision	5.45%	11.2%
Shaking	5.45%	9.6%
Loss of balance	5.4%	24.8%
Vaginal dryness	3.63%	3.2%
Muscle weakness	3.6%	13.6%
Slurred speech	3.6%	21.6%
Headache	3.6%	12.8%
Vomiting	0%	5.6%
Hallucinations	0%	8.8%

## Data Availability

The data that support the findings of this study are available on request from the corresponding author. The data are not publicly available due to privacy or ethical restrictions.
